# Effects of simulated digestion on the phenolic composition and antioxidant activity of different cultivars of lychee pericarp

**DOI:** 10.1186/s13065-019-0544-4

**Published:** 2019-03-09

**Authors:** Qingzhu Zeng, Zhuohui Xu, Mingrui Dai, Xuejiao Cao, Xiong Xiong, Shan He, Yang Yuan, Mingwei Zhang, Lihong Dong, Ruifen Zhang, Dongxiao Su

**Affiliations:** 1 School of Chemistry and Chemical Engineering, Guangzhou University, Guangzhou, 510006 People’s Republic of China; 2 Guangdong Key Laboratory of Agricultural Products Processing, Sericultural & Agri-Food Research Institute, Guangdong Academy of Agricultural Sciences, Guangzhou, 510610 People’s Republic of China

**Keywords:** Phenolic, HPLC, Lychee, Antioxidant activity, Simulated digestion

## Abstract

**Background:**

Lychee pericarp is rich in phenolic and has good antioxidant activity. The effects of simulated gastric (SGF) and intestinal fluid (SIF) digestion on the contents, composition, and antioxidant activities of the phenolic substances in the pericarp of different lychee cultivars (cv *Jizui*, *Lizhiwang*, *Guiwei*, *Yuhe*, *Nuomici* and *Guihong*) were investigated.

**Results:**

Compared with distilled water (DW) treatment, the total phenolic content (TPC) and total flavonoid content (TFC) in the pericarp of different lychee cultivars decreased after SGF digestion; especially, the TFC in “*Lizhiwang*” decreased by 41.5%. The TPC and TFC of lychee pericarp also decreased after SIF digestion. However, the TPC in “*Jizui*”, “*Guiwei*” and “*Yuhe*” increased. The SGF and SIF also had different effects on the FRAP and ABTS antioxidant activities of different lychee cultivars. The SGF digestion decreased the ABTS antioxidant capacity of lychee pericarp but enhanced the FRAP value of some lychee cultivars. However, the SIF digestion decreased the FRAP antioxidant activity of different lychee cultivar pericarps but enhanced the ABTS antioxidant capacity of lychee. The HPLC results showed that lychee pericarp had relatively high contents of procyanidin B2 and procyanidin A2. After SIF digestion, caffeic acid and isoquercitrin could not be detected in any of the lychee varieties. However, quercetin-3-rutinose-7-rhamnoside and isoquercitrin were increased after SGF digestion.

**Conclusions:**

Lychee pericarp could be used as an inexpensive functional food ingredient.

## Background

Lychee is a kind of fruit which is beneficial to human health [1]. It has brightly colored skin, translucent and congealed flesh, and a sweet and delicious taste, so it is very popular all over the world [2, 3]. Lychee is widely cultivated in tropical and subtropical countries [4], including China, India, Thailand, Vietnam and America. Among these countries, China has the highest yield and largest planting area.

In China, the commercial lychee cultivars are mainly “*Heiye*”, “*Feizixiao*”, “*Huaizhi*”, “*Guiwei*”, “*Baitangying*”, “*Baila*”, “*Jizui*”, “*Yuhe*” and “*Nuomici*”. The content of phenolic compounds in the lychee pericarp of these cultivars is not only determined by the type of plant, but also genetics, maturity and climatic conditions [5]. Su et al. [6] has shown that the total phenolic content in citrus peel is about 10–30 mg/g. The TPC of lychee pericarp was about 51–102 mg/g [2], which was higher than lotus leaves [6] and grape skins [7].

Lychee pericarp is rich in phenolic substances, such as epicatechin, procyanidins, cyanidin-3-glucoside, and quercetin-3-rutinoside [8]. The structures of eight phenolic compounds, including 2-(2-hydroxyl-5-(methoxycarbonyl) phenoxy) benzoic acid, kaempferol, isolariciresinol, stigmasterol, butylated hydroxytoluene, 3,4-dihydroxyl benzoate, methyl shikimate and ethyl shikimate, were confirmed by NMR and MS [9]. It has been proven that these phenolics have a strong scavenging ability and antioxidant capacity [10–14]. Not only that, but lychee pericarp, as a medicinal material, also has the capacity to dehumidify and stop dysentery and hemostasis, which reduces blood lipids, and has anti-cardiovascular disease [15] and anti-cancer [16–18] effects.

Lychee pericarp accounts for about 15% [19] of the total weight of fresh lychee. If these lychee pericarps are discarded directly, it will inevitably lead to a waste of resources [20]. The pericarp of lychee cannot be eaten directly, although the extraction of active substance from lychee pericarp, used as edible or medicinal ingredients, has great application prospects.

Phenolic substances of lychee pericarp extracts would be affected by the gastrointestinal tract before they are absorbed. The gastric digestion and intestinal digestion would have different effects on the composition and content of phenolic profiles, and thus change their antioxidant activity [21, 22]. After simulated digestion in vitro, previous studies proved that the content of phenolic substances and its antioxidant activity will increase [23, 24], while others found it will decrease [25]. There are few reports on the effects of simulated digestion on the phenolic compounds and antioxidant activities of lychee pericarp. Therefore, the aim of the present study is to compare the influence of SGF and SIF digestion on the composition and content of phenolic substances of six varieties of lychee pericarps, and to explore the change of phenolic compounds caused by simulated digestion on antioxidant activity.

## Results

### Effects of simulated digestion on the TPC of different commercial varieties of lychee pericarp

**Fig. 1:**
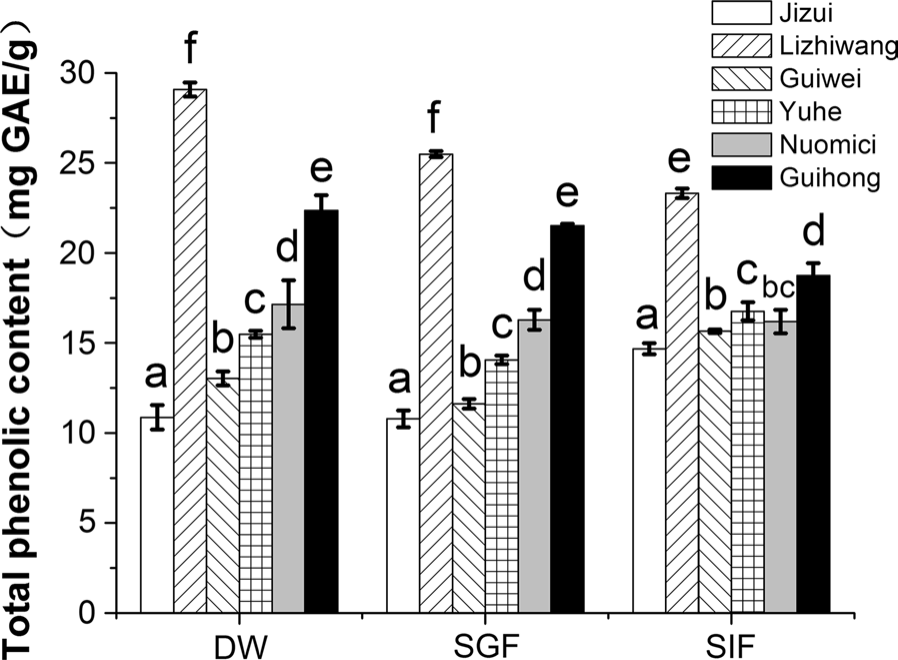
Effects of simulated digestion on total phenolic content in different varieties of lychee pericarp. Values with different letters within one extraction method are significantly different. *DW* distilled water extraction, *SGF* simulated gastric fluid extraction, *SIF* simulated intestinal fluid extraction

The effects of different digestion treatments on the TPC of the lychee pericarp of different varieties are shown in Fig. 1. The TPC of lychee “*Lizhiwang*” was the highest, followed by “*Guihong*”. In the DW extraction group, the TPC of “*Lizhiwang*” was 1.7-fold higher than that of “*Jizui*”, which had the least TPC (*p* < 0.05), and the commercial variety, “*Nuomici*”, was 0.6-fold higher than “*Jizui*” (*p* < 0.05), which is also a commercial variety. The TPC in the pericarp of different lychee cultivars was significantly different after distilled water extraction and SGF treatment (*p* < 0.05). After SGF digestion, the TPC in the pericarp of different lychee varieties was lower than that of the DW extraction group. However, the TPC of “*Jizui*”, “*Guiwei*” and “*Yuhe*” increased after SIF digestion, compared with the DW extraction group (*p* < 0.05). Finally, compared with DW, the TPC of the “*Lizhiwang*”, “*Nuomici*” and “*Guihong*” varieties decreased after the extraction of SGF and SIF.

### Effects of simulated digestion on the TFC of different commercial varieties of lychee pericarp

**Fig. 2:**
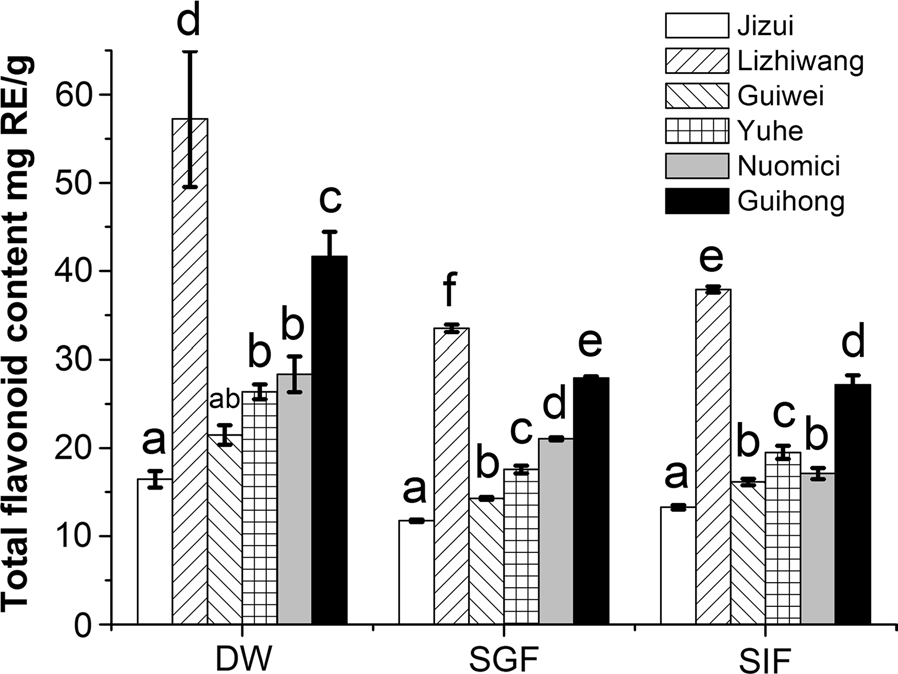
Effects of simulated digestion on total flavonoid content in different varieties of lychee pericarp. Values with different letters within one extraction method are significantly different. *DW* distilled water extraction, *SGF* simulated gastric fluid extraction, *SIF* simulated intestinal fluid extraction

The effects of different digestion treatments on the TFC of lychee pericarp of different varieties are shown in Fig. 2. In the DW extraction group, the TFC of “*Lizhiwang*” was 2.5-fold higher than that of “*Jizui*”, which had the least TFC (*p* < 0.05), and the commercial variety, “*Nuomici*”, was 0.7-fold higher than “*Jizui*” (*p* < 0.05), which is also a commercial variety. After DW extraction and SGF digestion, the ranking was “*Lizhiwang*” > “*Guihong*” > “*Nuomici*” > “*Yuhe*” > “*Guiwei*” > “*Jizui*”. After simulated intestinal digestion, the ranking was “*Lizhiwang*” > “*Guihong*” > “*Yuhe*” > “*Nuomici*” > “*Guiwei*” > “*Jizui*”. After SGF digestion, the TFC in the pericarp of different lychee cultivars was significantly different (*p* < 0.05). However, there was no significant difference between “*Guiwei*” and “*Nuomici*” after SIF digestion (*p* > 0.05). The TFC in the pericarp of different lychee varieties, after SGF or SIF treatments, were lower than those of the DW group. Among the different treatments, “*Lizhiwang*” had the highest TFC, and “*Guihong*” followed. Among the different treatments, the content ranking of the TFC and TPC of lychee pericarp was completely consistent.

### Effects of simulated digestion on the FRAP antioxidant capacity of different commercial varieties of lychee pericarp

**Fig. 3:**
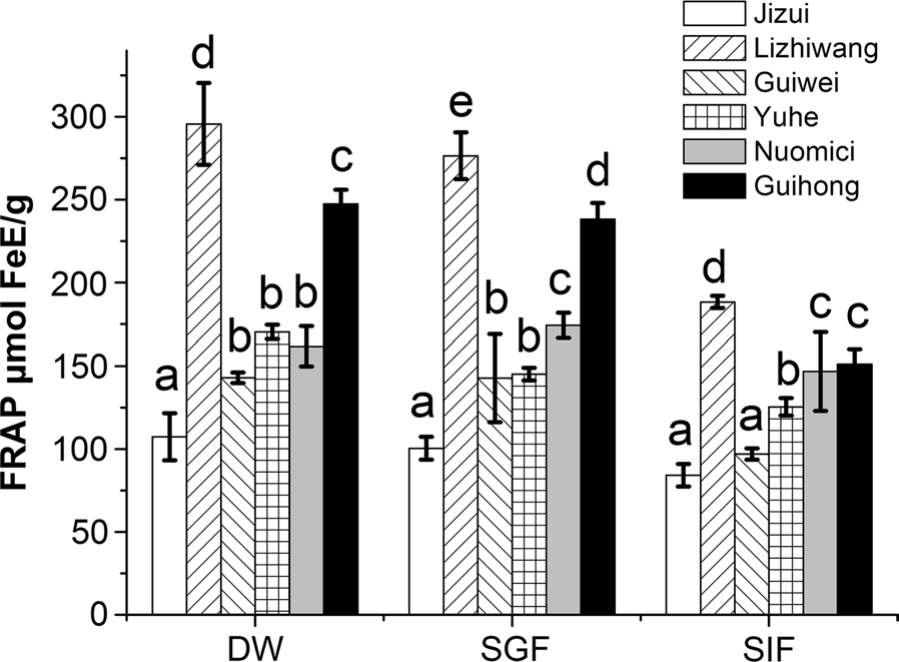
Effects of simulated digestion on FRAP antioxidant capacity in different varieties of lychee pericarp. Values with different letters within one extraction method are significantly different. *DW* distilled water extraction, *SGF* simulated gastric fluid extraction, *SIF* simulated intestinal fluid extraction

The effects of different digestion treatments on the FRAP antioxidant capacity of the lychee pericarp of different varieties are shown in Fig. 3. There was no significant difference (*p* > 0.05) between “*Guiwei*” and “*Yuhe*” after DW extraction and SGF digestion. However, after SIF digestion, there was a significant difference (*p* < 0.05) between “*Guiwei*” and “*Yuhe*”. After SGF digestion, the FRAP antioxidant capacity of “*Nuomici*” was stronger than that of the DW extraction group. Finally, after DW extraction, SGF and SIF digestion, the FRAP antioxidant capacity of “*Lizhiwang*” and “*Guihong*” was higher than that of other lychee cultivars (*p* < 0.05).

### Effects of simulated digestion on the ABTS antioxidant capacity of different commercial varieties of lychee pericarp

**Fig. 4:**
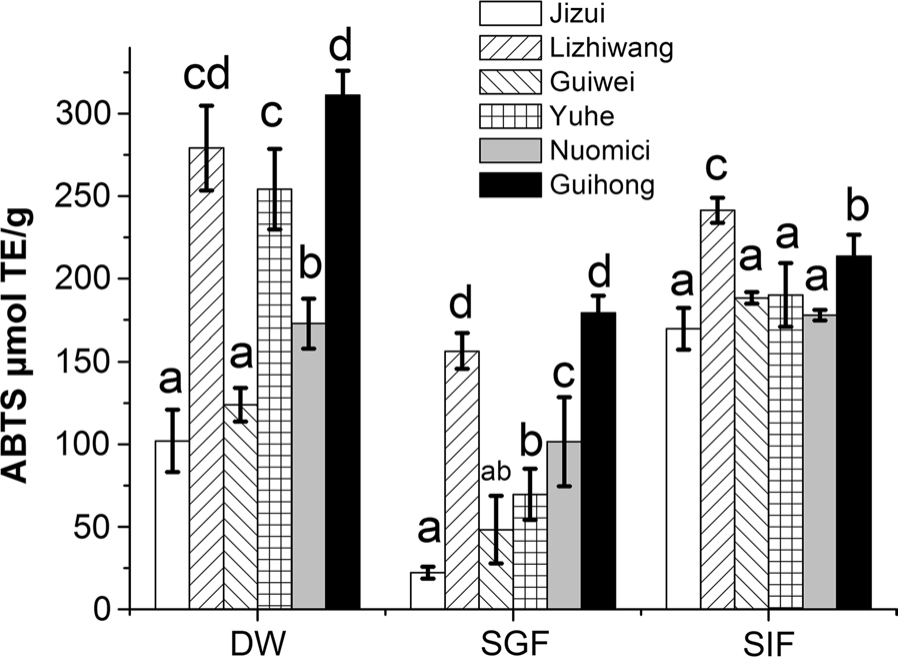
Effects of simulated digestion on ABTS antioxidant capacity in different varieties of lychee pericarp. Values with different letters within one extraction method are significantly different. *DW* distilled water extraction, *SGF* simulated gastric fluid extraction, *SIF* simulated intestinal fluid extraction

The effects of different digestion treatments on the ABTS antioxidant capacity of the lychee pericarp of different varieties are shown in Fig. 4. There was no significant difference (*p* > 0.05) in the ABTS antioxidant capacity between “*Jizui*” and “*Guiwei*”. After SGF digestion, the ABTS antioxidant capacity of all lychee cultivars was weaker than that of the DW extraction group (*p* < 0.05). However, after SIF extraction, the ABTS antioxidant activity of “*Jizui*” and “*Guiwei*” was stronger than that of the DW extraction group (*p* < 0.05). After SIF digestion, the ABTS antioxidant capacity of lychee pericarp of all varieties was stronger than that following SGF digestion (*p* < 0.05). Finally, after DW extraction, SGF and SIF digestion, the ABTS antioxidant capacity of “*Lizhiwang*” and “*Guihong*” was higher than that of other lychee cultivars (*p* < 0.05). However, both in the DW extraction group and SGF digestion treatment group, the ABTS antioxidant activity of “*Guihong*” was stronger than that of “*Lizhiwang*”.

### Effects of simulated digestion on the phenolic composition of different commercial cultivars of lychee pericarp

The effects of simulated digestion on the monomeric phenolics of different varieties of lychee pericarp, detected by HPLC, are shown in Table 1. Two phenolic acids (caffeic acid and ferulic acid), four procyanidin (procyanidin B2, epicatechin, A-type procyanidin trimer and procyanidin A2) and two flavonols (quercetin-3-rutinose-7-rhamnoside and isoquercitrin) were detected in lychee pericarp. The HPLC results showed that the content of caffeic acid after DW extraction was in the order of *Lizhiwang*, *Guihong*, *Guiwei*, *Nuomici*, *Yuhe*, and *Jizui*. However, after SGF digestion, the sequence became *Lizhiwang*, *Guihong*, *Nuomici*, *Yuhe*, *Guiwei*, and *Jizui*, which was consistent with the TPC. However, caffeic acid in the lychee pericarp of all six lychee cultivars could not be detected after SIF digestion. The change in ferulic acid was different with caffeic acid. The ferulic acid in “*Guihong*” could not be detected after SGF digestion. After DW extraction, the content order of procyanidin B2, epicatechin, A-type procyanidin trimer and procyanidin A2 content in different varieties was inconsistent. After DW extraction, the content of A-type procyanidin trimer was the highest in “*Jizui*” and significantly different from that of the other varieties (*p* < 0.05). After SGF digestion, the A-type procyanidin trimer in “*Jizui*” could not be detected. After SIF digestion, the A-type procyanidin trimer in “*Guiwei*”, “*Yuhe*”, “*Nuomici*” and “*Guihong*” could not be detected. The content of Procyanidin A2 in “*Lizhiwang*” was the highest among the six cultivars, and it was significantly different from that of the other cultivars (*p* < 0.05). There was no significant difference in the content of quercetin-3-rutinose-7-rhamnoside between “*Jizui*” and “*Nuomici*” (*p* > 0.05) after DW extraction, but a significant difference was observed after SGF digestion (*p* < 0.05). After SIF digestion, the content of isoquercitrin in the pericarp of six lychee cultivars could not be detected. Based on the obtained results, it was found that, after DW extraction, the order of the TPC in different varieties of lychee pericarp, measured by the chemical method, was consistent with that determined by HPLC, and the order was as follows: “*Lizhiwang*” > “*Guihong*” > “*Nuomici*” > “*Yuhe*” > “*Guiwei*” > “*Jizui*”. After SGF digestion, the order of TFC in different varieties of lychee pericarp, measured by the chemical method, was consistent with that determined by HPLC, and the order was as follows: “*Lizhiwang*” > “*Guihong*” > “*Nuomici*” > “*Yuhe*” > “*Guiwei*” > “*Jizui*”.

**Table 1:** Effects of simulated digestion on monomeric phenolic in different varieties of lychee pericarp by HPLC–DAD

Mono phenolic (mg/g DW)	Jizui	Lizhiwang	Guiwei	Yuhe	Nuomici	Guihong
*Caffeic acid*						
DW	0.10 ± 0.01^a^	0.99 ± 0.04^c^	0.24 ± 0.03^a^	0.16 ± 0.07^a^	0.21 ± 0.05^a^	0.54 ± 0.17^b^
SGF	0.07 ± 0.00^a,^*	0.79 ± 0.03^e,^*	0.25 ± 0.06^b^	0.28 ± 0.01^b,^*	0.50 ± 0.12^c,^*	0.69 ± 0.02^d^
SIF	ND^a,^*	ND^a,^*	ND*	ND^a,^*	ND^a,^*	ND^a,^*
*Ferulic acid*						
DW	0.35 ± 0.02^a^	3.39 ± 0.18^e^	0.83 ± 0.05^b^	1.07 ± 0.33^b^	1.42 ± 0.20^c^	1.74 ± 0.03^d^
SGF	0.25 ± 0.05^b,^*	0.09 ± 0.06^a,^*	0.38 ± 0.04^c,^*	0.40 ± 0.08^ac,^*	1.32 ± 0.07^d^	ND^a,^*
SIF	0.17 ± 0.07^a,^*	0.87 ± 0.21^d,^*	0.34 ± 0.06^b,^*	0.06 ± 0.01^a,^*	0.54 ± 0.05^c,^*	0.59 ± 0.01^c,^*
*Procyanidin B2*						
DW	1.14 ± 0.19^a^	9.42 ± 0.41^e^	3.09 ± 0.28^c^	2.49 ± 0.11^b^	2.04 ± 0.49^b^	5.56 ± 0.35^d^
SGF	0.73 ± 0.10^a,^*	7.05 ± 0.28^e,^*	2.63 ± 0.31^c^	2.14 ± 0.17^b,^*	2.77 ± 0.02^c,^*	6.19 ± 0.11^d,^*
SIF	0.70 ± 0.03^a,^*	1.47 ± 0.29^c,^*	0.49 ± 0.02^a,^*	0.48 ± 0.04^a,^*	0.40 ± 0.04^a,^*	1.14 ± 0.08^b,^*
*Epicatechin*						
DW	0.16 ± 0.07^a^	4.48 ± 0.59^d^	2.25 ± 0.04^b^	1.81 ± 0.11^b^	3.23 ± 0.31^c^	4.50 ± 0.36^d^
SGF	0.87 ± 0.13^a,^*	3.60 ± 0.34^c^	1.39 ± 0.28^a,^*	1.39 ± 0.22^a,^*	2.97 ± 0.49^b^	3.85 ± 0.15^c,^*
SIF	0.39 ± 0.05^a,^*	2.19 ± 0.83^c,^*	0.21 ± 0.00^a,^*	0.06 ± 0.02^a,^*	1.19 ± 0.36^b,^*	1.98 ± 0.07^c,^*
*A*-*type procyanidin trimer*						
DW	1.10 ± 0.11^d^	0.81 ± 0.07^c^	0.07 ± 0.01^a^	0.03 ± 0.00^a^	0.38 ± 0.01^b^	0.38 ± 0.16^b^
SGF	ND^a,^*	1.15 ± 0.23^b^	0.16 ± 0.08^a,^*	0.02 ± 0.00^a,^*	0.05 ± 0.05^a,^*	0.17 ± 0.08^a,^*
SIF	0.15 ± 0.01^a,^*	2.09 ± 0.40^b,^*	ND^a^	ND^a,^*	ND^a,^*	ND^a,^*
*Procyanidin A2*						
DW	1.50 ± 0.09^a^	3.76 ± 0.08^d^	1.36 ± 0.21^a^	1.97 ± 0.08^b^	1.32 ± 0.13^a^	2.58 ± 0.18^c^
SGF	1.01 ± 0.10^a,^*	2.89 ± 0.80^c^v	1.39 ± 0.02^a^*	1.92 ± 0.21^ab^	1.22 ± 0.18^a^	2.52 ± 0.54^bc^
SIF	0.94 ± 0.04^a^*	2.22 ± 0.34^b^*	0.87 ± 0.07^a^	0.83 ± 0.00^a^*	1.66 ± 0.28^a^	1.91 ± 0.13^a^
*Quercetin*-*3*-*rutinose*-*7*-*rhamnoside*						
DW	0.26 ± 0.02^a^	2.00 ± 0.06^c^	0.64 ± 0.10^b^	0.81 ± 0.09^b^	0.40 ± 0.18^a^	0.81 ± 0.14^b^
SGF	0.29 ± 0.04^a^	2.72 ± 0.14^e^*	0.54 ± 0.05^b^	0.82 ± 0.14^c^	0.70 ± 0.03^bc^*	1.36 ± 0.09^d^
SIF	1.06 ± 0.00^b^*	0.46 ± 0.13^a^*	0.36 ± 0.08^a^*	0.49 ± 0.03^a^*	0.77 ± 0.01^b^*	1.09 ± 0.02^b^
*Isoquercitrin*						
DW	0.32 ± 0.06^a^	1.42 ± 0.09^d^	0.35 ± 0.04^a^	0.50 ± 0.04^b^	0.41 ± 0.06^ab^	0.71 ± 0.05^c^
SGF	0.34 ± 0.04^a^	1.30 ± 0.16^d^	0.30 ± 0.06^a^	0.57 ± 0.11^b^	0.46 ± 0.08^ab^	0.94 ± 0.09^c,^*
SIF	ND^a,^*	ND^a,^*	ND^a,^*	ND^a,^*	ND^a,^*	ND^a,^*

Values expressed as mg/g DW

*ND* not detected, *DW* distilled water extraction, *SGF* simulated gastric fluid digestion, *SIF* simulated intestinal fluid digestion

Values not sharing a common letter within the same row indicate a significant difference (*p* < 0.05). In the same monomer phenolic and the same variety

* Stands for significant difference with DW (*p* < 0.05). mean ± SD, n = 3. The content of A-type procyanidin trimer was calculated by the standard curve of procyanidin A2

**Fig. 5:**
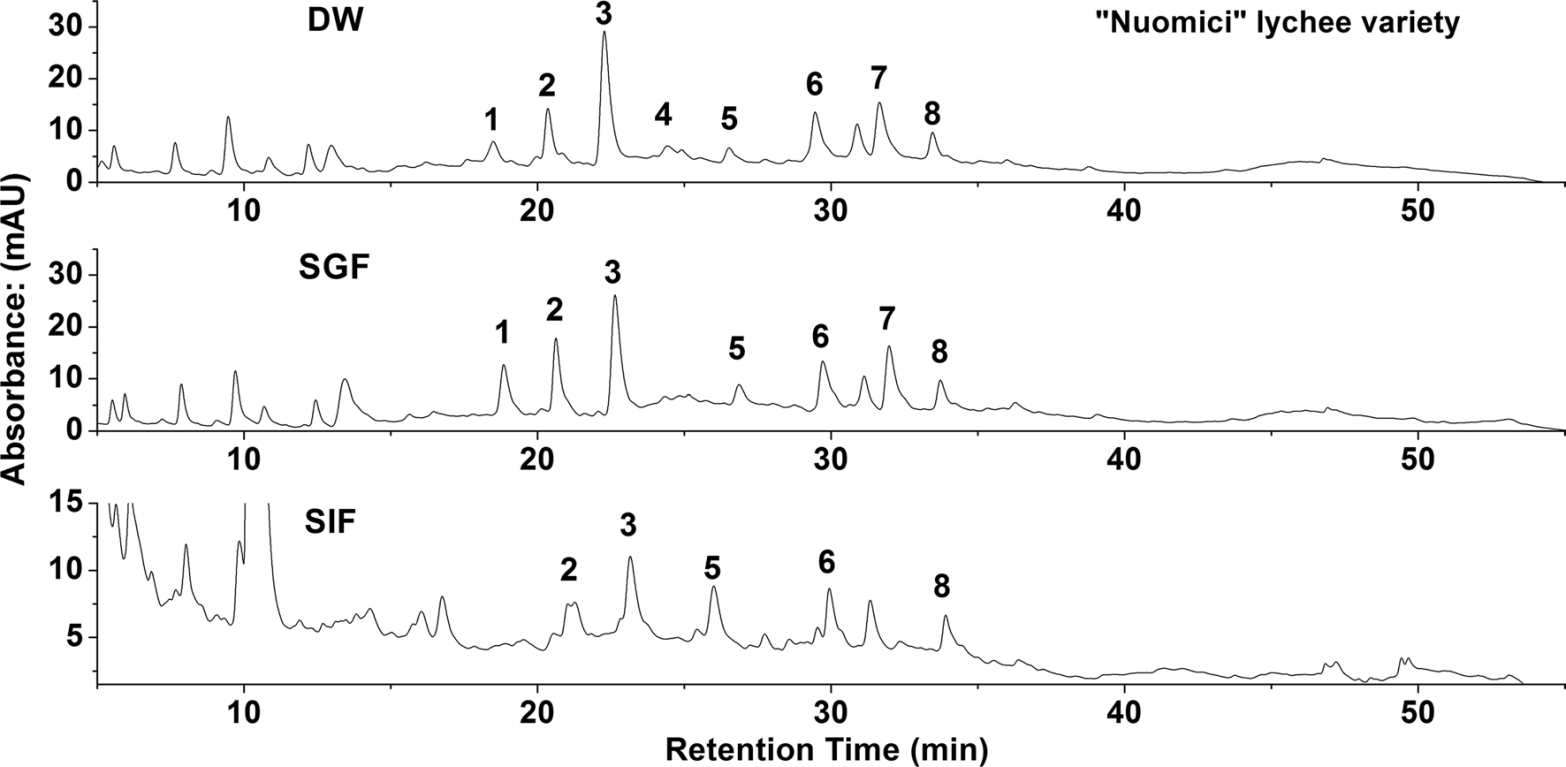
Effects of simulated digestion on the phenolic composition in “*Nuomici*” lychee variety by HPLC. *DW* distilled water extraction, *SGF* simulated gastric fluid extraction, *SIF* simulated intestinal fluid extraction

The “*Nuomici*” variety was a representative of commercial products. The composition and content of the main phenolic compounds in the pericarp of “*Nuomici*” for DW extraction, SGF and SIF digestion were analyzed using HPLC, as shown in Fig. 5 and Table 1. By comparing the retention time of the chromatographic peaks with the standard, it was determined that peak nos. 1, 2, 3, 4, 5, 6, 7 and 8 were caffeic acid, procyanidin B2, epicatechin, A-type procyanidin trimer, quercetin-3-rutinose-7-rhamnoside, ferulic acid, isoquercitrin and procyanidin A2, respectively. Peak no. 4, A-type procyanidin trimer, was virtually undetectable after SGF digestion. Peak nos. 1 and 7, caffeic acid and isoquercitrin, could not be detected after SIF digestion. However, after SGF digestion, the content of caffeic acid was significantly higher than that of the DW extraction group (*p* < 0.05), but it could not be detected after SIF digestion (*p* < 0.05). After SGF digestion, the content of ferulic acid was not noticeably decreased, compared with that of the DW extraction group, but the content was significantly decreased after digestion with SIF (*p* < 0.05). After SGF digestion, the content of procyanidin B2 was significantly higher than that of the DW extraction group (*p* < 0.05). However, after SGF digestion, the content of epicatechin was reduced, and the content was significantly lower after SIF digestion than that of the DW extraction group (*p* < 0.05). Similar to epicatechin, the content of A-type procyanidin trimer was lower after SGF digestion than that of the DW extraction group, but it could not be detected after SIF digestion (*p* < 0.05). After SGF digestion, the content of quercetin-3-rutinose-7-rhamnoside and isoquercitrin were increased, compared to that of the DW extraction group, but they could not be detected after SIF digestion (*p* < 0.05). Taken together, compared with the DW extraction group, the content of the monomer phenolic composition of lychee had almost completely disappeared after SGF or SIF extraction, but there were some increases after SGF digestion, and caffeic acid, procyanidin B2, and quercetin-3-rutinose-7-rhamnoside increased significantly (*p* < 0.05).

## Discussion

Fruit pericarp is rich in phenolic substances, which have good antioxidant, anti-inflammatory and antibacterial activities [26]. Phenolic compounds play important roles in antioxidants [27]. However, the content of phenolic compounds is related to the growth environment and genes of fruit. The same plant growth in different environments or of different varieties will lead to different phenolic content, composition and antioxidant activity [28]. Li et al. [29] studied the phenolic content, composition and antioxidant activity in the pericarp of 10 lychee cultivars and found that the above indicators of different lychee cultivars varied greatly.

Digestion also affects the content, composition and antioxidant capacity of phenolic compounds. A further study digested 10 different walnut varieties in vitro and found that the TPC and antioxidant capacity of walnut decreased in varying degrees. However, the present study found that the phenolic content in the pericarp of different lychee cultivars did not decrease completely after simulated digestion in vitro. The TPC of “*Jizui*”, “*Guiwei*” and “*Yuhe*” lychee cultivars increased after intestinal digestive fluid treatment. Previous studies have shown that SGF releases phenolics from fruit [30, 31], which was similar to the result of the present study. Su et al. [32] studied lotus leaves at different stages using a digestion model in vitro. The results showed that the content of phenolic substances in lotus leaves at different growth stages increased after SIF digestion, which was similar to the results of this study. The TFC in pericarp of different lychee cultivars after SGF and SIF digestion was lower than that of the DW extraction group. Ovando-Martnez et al. [33] reported that the TFC of red chiltepin decreased after digestion in simulated gastrointestinal fluid, which was consistent with the present study.

Lychee pericarps have a good FRAP reducing ability and ABTS radical scavenging capacity. Previous studies have shown that antioxidant activity was positively related to the TPC and TFC [26, 34–36]. Lychee pericarp contains abundant phenolic substances, including phenolic acids, flavonoids and proanthocyanidins, which indirectly prove that lychee pericarp could have strong antioxidant and anti-free radical activities. Further analysis, the strength of the antioxidant activity depends on the number of hydroxyl groups in the chemical structure of the phenolic substances [16], that is, the greater the number of hydroxyl groups, the stronger the antioxidant capacity. The antioxidant activities of different lychee pericarps, treated by SGF or SIF, were evaluated using the FRAP and ABTS methods. The ABTS antioxidant capacity in the pericarp of 6 lychee cultivars decreased after SGF digestion, but the FRAP antioxidant capacity of some lychee cultivars increased. The FRAP antioxidant capacity in “*Lizhiwang*” after SGF digestion was stronger than that after SIF digestion, but the opposite was the case for ABTS. Su et al. [32] evaluated the FRAP and ABTS antioxidant activities in lotus leaves at different growth stages by SGF or SIF treatment. It was found that the FRAP antioxidant capacity in lotus leaves, at different growth stages, after SGF digestion, was stronger than that after SIF digestion, but the opposite was the case for ABTS. The results were similar to those of this study. The FRAP antioxidant capacity in the pericarp of most lychee cultivars decreased after SGF digestion and decreased completely after SIF digestion. The ABTS antioxidant capacity of the pericarp of some lychee cultivars increased after SIF digestion and decreased completely after SGF digestion. The reason may be related to the release of phenolics under different pH values using different treatment methods, or to the reaction between phenolic substances and proteinase in specific circumstances, producing different results. Tagliazucchi et al. [23] reported that the pH value and phenolic content during digestion affected the antioxidant activity. However, the reasons and mechanisms still need further study.

Lychee pericarp is rich in natural antioxidant phenolics [2]. Eight phenolic compounds were isolated from lychee pericarp by Jiang et al. [9]. Their structures were confirmed by NMR and MS. Zhang et al. [37] identified 6 phenolic compounds from lychee pericarp by HPLC–MS. Eight phenolic compounds were tentatively identified by HPLC–DAD in the present study. The HPLC results showed that the monomer phenolic of lychee pericarp was significantly affected by digestion methods. After SIF digestion, caffeic acid and isoquercitrin were not detected in the pericarp of any of the lychee varieties. It is possible that the phenolic combined with proteins, carbohydrates or lipids to form complexes [38], which were mostly linked by non-covalent bonds, such as hydrogen bonds [39]. This would slow down the decline of phenolic content. In general, compared with the DW extraction group, the monomer phenolic content showed a downward trend after SGF digestion, and the downward trend was more severe after SIF digestion. SGF digestion is carried out under acidic conditions, because these phenolic substances have been proven to be more stable under acidic conditions in previous studies, and the lower the pH, the better the stability [40]. The phenolic substances decreased more in the SIF digestion process, probably because the structure of phenolic in lychee was more phenolic hydroxyl, acidic, unstable and easily degraded than other substances in an alkaline environment [41]. After SGF digestion, the content of Procyanidin B2 in “*Jizui*”, “*Lizhiwang*”, “*Guiwei*” and “*Yuhe*” decreased, compared with DW. Previous studies reported that the content of proanthocyanidin B2 in apple decreased in gastric digestion. After SIF digestion, the content of proanthocyanidin B2 in the pericarp of all lychee cultivars decreased, compared with DW. Bouayed et al. [42] and Zhu et al. [43] found that the content of proanthocyanidin B2 decreased in the intestinal environment. They believe that procyanidin B2 may produce other compounds in intestinal fluid. Procyanidin B2 may be degraded in intestinal fluid, but the specific degradation pathway and degradation products need to be further confirmed.

## Experimental section

### Materials

Six varieties of fresh lychee fruit, including “*Jizui*”, “*Lizhiwang*”, “*Guiwei*”, “*Yuhe*”, “*Nuomici*” and “*Guihong*”, about 5 kg for each variety were purchased from local farmers markets. These lychee varieties were carefully examined and identified by professor Mingwei Zhang from the Guangdong Academy of Agricultural Sciences. Commercially mature, bright red and uniform size lychees were chose for the following experiment. All the above lychee pericarp was stripped manually and rinsed with tap water, then dried in the electro thermal constant temperature air drying oven (DGG-9070A, Shanghai Senxin Experimental Instrument Co., Ltd., Shanghai China) at 60 °C until the moisture content less than 8%. After that, approximately 70 g of dried lychee pericarp of each variety were obtained. The dried lychee pericarp samples were crushed by a mechanical grinder (WK-400B, Shandong Qingzhou Jingcheng Machinery Co., Ltd. Qingzhou China), and then passed through a 40-mesh sieve. Finally, they were packed in sealed bags and kept in dryer at room temperature avoiding light. Lychee pericarp powder of each variety were mixed evenly and weighed randomly for the following study.

Gallic, rutin, and Trolox(6-hydroxy-2,5,7,8-tetramethy lchroman-2-2carboxylic acid)were purchased from Shanghai Yuanye Biotechnology Co., Ltd. (Shanghai, China). ABTS [2,2′-azinobis-(3-ethylbenzthiazoline-6-sulphonate)], TPTZ (2,4,6-tripyridyl-*s*-triazine) and Ferrous sulphate were purchased from Xiya Reagent Co., Ltd (Chengdu China). Folin-Ciocalteu reagent were purchased from Macklin Reagent Co., Ltd (Shanghai China).

### Simulated digestion in vitro

Simulated gastric fluid digestion (SGF) and simulated intestinal digestion (SIF) were prepared according to the United States Pharmacopeia and Fu et al. [44].

The preparation of SGF is as follows: 2.00 g of sodium chloride and 3.20 g of pepsin (BR, 3000USPu/mg activity units, Macklin Reagent Co., Ltd., Shanghai, China) were added to 950 mL of distilled water and 7.0 mL of concentrated hydrochloric acid. The mixture was stirred and oscillated to fully dissolve the ingredients. The pH was adjusted to 1.2 with hydrochloric acid, while stirring with a magnetic stirrer. Finally, the solution was filled to a constant volume of 1000 mL and stored overnight until use.

The treatment with SGF is as follows. Three replication samples of each variety were weighed at 1.00 ± 0.01 g and added to 50 mL plastic centrifuge tubes. Then, 30 mL of SGF was added and mixed with a whirlpool mixer (XW-80A, Linbeier Instrument Manufacturing Co., Ltd., Jiangsu, China). Next, the tube was incubated in a water bath thermostat oscillator (THZ-82, Jintan Huaou Experimental Instrument Factory, Jiangsu, China) at 37 °C and 120 r/min for 120 min. Finally, the tube was centrifuged (GL-2050MS, Lu Xiangyi Centrifuge Instrument Co., Ltd., Shanghai, China) at 5000 r/min for 10 min to collect the supernatant. The supernatant was collected in a 10 mL centrifuge tube and stored in a freezer at − 20 °C until use.

The preparation of SIF is as follows: Potassium dihydrogen phosphate (6.80 g) was added to 250 mL of distilled water. Then, 190 mL of 0.2 mol/L sodium hydroxide solution and 400 mL of distilled water were added, and the pH was adjusted to 7.5 ± 0.1 with sodium hydroxide solution or hydrochloric acid solution. Next, 10.00 g of pancreatin (BR, 4000 USPu/mg activity units, Yuanye Biotechnology Co., Ltd., Shanghai, China) was added to the solution. Finally, the fluid was transferred to a 1000 mL volumetric flask with a fixed capacity.

The treatment with SIF is as follows. The samples, three replication of each variety, were weighed (1.00 ± 0.01 g) and added to 50 mL plastic centrifuge tubes and mixed with the previously added 30 mL of SIF. The solution was mixed and incubated, as in SGF digestion. After centrifugation, the supernatant was collected and stored in a freezer at − 20 °C until use.

### Distilled water extraction

The lychee pericarp powder samples, three replication of each variety, were weighed (1.00 ± 0.01 g), and added to 50 mL plastic centrifuge tubes and mixed with 30 mL of distilled water (DW). The following processing steps were similar to the process of SGF digestion.

### Determination of total phenolic content

The Folin–Ciocalteu (FC) colourimetric method was used to determine the total phenolic content in different lychee pericarps, following the method reported by Hossain and Rahman [45]. The diluted sample (250 μL) was added to 1.0 mL of distilled water and 250 μL of Folin–Ciocalteu reagent and allowed to stand for 6 min, after mixing with a whirlpool mixer. Then, 2.70 mL of sodium carbonate solution, with a concentration of 7% and 2.00 mL of distilled water, was added to the solution. Next, the reaction was carried out in a dark room at room temperature for 90 min. The absorbance at 760 nm was measured with a UV–Vis spectrophotometer (UV-2100, Beijing Rayleigh Analytical Instrument Co., Ltd., Beijing, China). Gallic acid was used as the standard, and the total phenolic contents were expressed as mg gallic acid (calibration range of 50–250 μg/mL, correlation coefficient = 0.9985) equivalent (GAE)/g. The results were carried out in triplicate for each variety and presented as mean ± SD.

### Determination of total flavonoid content

The determination of the total flavonoid content (TFC) in lychee pericarp refers to Hossain and Rahman [45]. The diluted sample (600 μL) was added to 180 μL of sodium nitrite solution (m: v = 5%) and 3.00 mL of distilled water and allowed to stand for 6 min, after mixing with a vortex mixer. Then, 360 μL of aluminum chloride hexahydrate solution, with a mass concentration of 10%, was added, and the reaction took place at room temperature for 5 min. Then, 1.20 mL of 1 mol/L sodium hydroxide solution was added. Finally, 0.66 mL of distilled water was added to make up the remaining 6.00 mL. The absorbance at 510 nm was measured. Rutin (calibration range of 200–1000 μg/mL, R^2^ = 0.9962) was used as the standard, and the total flavonoid contents were expressed as mg rutin equivalent (RE)/g. The results were determined in triplicate for each variety and presented as mean ± SD.

### Determination of antioxidant capacity by the FRAP method

The specific measurement of the FRAP method refers to Thaipong et al. [46]. Briefly, 0.3 mL of the diluted sample and 2.7 mL of the FRAP working solution were mixed, placed in the dark and allowed to react at room temperature for 30 min. The absorbance at 593 nm was measured. The FRAP antioxidant capacity was expressed as μmol ferrous ion (calibration range of 0.15–1.5 μmol/mL, R^2^ = 0.9983) equivalent (FeE)/g. The results were presented as mean ± SD gained from three replication for each variety.

### Determination of antioxidant capacity by the ABTS method

The specific determination the ABTS method refers to Thaipong et al. [46]. Briefly, 0.1 mL of the sample and 2.9 mL of the ABTS working fluid were mixed well with whirlpool oscillation and allowed to react for 6 min, before measurement at 734 nm. The ABTS antioxidant capacity was expressed as μmol trolox (calibration range of 0.1–0.6 μmol/mL, R^2^ = 0.9955) equivalent (TE)/g. The results were presented as mean ± SD acquired from three determination for each variety.

### Determination of phenolic composition by HPLC

The composition of the phenolic compounds in lychee pericarp extract was determined by a previously reported HPLC method [47, 48]. HPLC analysis was performed by an Agilent 1260 series system instrument (Agilent Technologies 1260 Infinity LC, CA) equipped with a four element pump (G1311C 1260 Quat Pump VL) delivery system, an automatic sampler (G1329B 1260ALS), and a DAD detector (G1315D DAD). Chromatographic separations were carried out on 250 mm * 4.6 mm, 5 μm Zorbax SB-C18 column (Agilent Technologies, Palo Alto, CA). HPLC–DAD analysis was performed at 30 °C, with a flow rate of 1.0 mL per min and an injection volume of 20 μL. Acetonitrile (A) and 0.4% glacial acetic acid (B) were used as a mobile phase composition. The gradient elution program was as follows: 0–40 min, A 5%–25%; 40–45 min, A 25%–35%; and 45–50 min, A 35%–50%. Chromatographic data was recorded at 280 nm. All solvents were of HPLC grade and filtered with a 0.45 µm filter disk. Prior to analysis, all of the samples were filtered through a 0.45 µm membrane filter. Milli-Q water (Millipore) was used throughout. The chromatographic peaks were tentatively identified according to the retention time of standard compounds, including caffeic acid (the calibration range of 2–200 μg/mL, with correlation coefficient 0.9998), procyanidin B2 (1–500 μg/mL, R^2^ = 0.9989), epicatechin (1–350 μg/mL, R^2^ = 0.9995), quercetin-3-rutinose-7-rhamnoside (3–500 μg/mL, R^2^ = 0.9999), ferulic acid (0.6–120 μg/mL, R^2^ = 0.9999), isoquercitrin (3–500 μg/mL, R^2^ = 0.9996) and procyanidin A2 (1–800 μg/mL, R^2^ = 0.9988), and the peak area was used for quantitative analysis. The results were expressed as mg/g of lychee pericarp. The results were presented as mean ± SD obtained from three replication for each variety [48].

### Statistical analysis

All analysis were conducted in triplicate, and the results were expressed as mean ± standard deviation. One-way analysis of variance (ANOVA) was performed using SPSS 24.0 statistical software, and a S–N–K test was used to compare the significant differences among the varieties. The significance level was *p *< 0.05. Significant differences between the different lychee varieties are represented by different lowercase letters. Origin 7.5 was used for mapping.

## Conclusions

The effects of simulated digestion in vitro on the TPC, TFC, FRAP and ABTS antioxidant activity in the pericarp of six lychee cultivars were studied. After SGF digestion, the TPC in the pericarp of different lychee varieties was lower than that of the DW extraction group. However, the TPC of “*Jizui*”, “*Guiwei*” and “*Yuhe*” increased after SIF digestion, compared with the DW extraction group. The TFC in the pericarp of different lychee varieties was lower than that of the DW group after both SGF and SIF digestion. However, the FRAP and ABTS antioxidant capacity of “*Lizhiwang*” and “*Guihong*” was higher than that of other lychee cultivars. The ABTS antioxidant capacity in the lychee pericarp of all varieties after SIF digestion was stronger than that after SGF digestion. Eight phenolic monomers were detected in lychee pericarp, including caffeic acid, procyanidin B2, epicatechin, A-type procyanidin trimer, quercetin-3-rutinose-7-rhamnoside, ferulic acid, isoquercitrin and procyanidin A2. The caffeic acid and isoquercitrin in the pericarp of six lychee cultivars could not be detected after SIF digestion. However, the quercetin-3-rutinose-7-rhamnoside and isoquercitrin were increased after SGF digestion. Extracorporeal SGF and SIF had different effects on the phenolic compounds in different varieties of lychee pericarp.

## Abbreviations

SGF: simulated gastric fluid
SIF: simulated intestinal fluid
DW: distilled water
TPC: total phenolic content
TFC: total flavonoid content
FRAP: ferric ion reducing antioxidant power
ABTS: 2,2′-azino-bis (3-ethylbenzothiazoline-6-sulfonic acid)
HPLC: high performance liquid chromatography
